# Dataset on the prevalence of tobacco smoking in men and women of selected countries whit difference human development

**DOI:** 10.1016/j.dib.2018.03.043

**Published:** 2018-03-16

**Authors:** Mina Riahi, Ali Akbar Mohammadi, Hosein Rohani, Mohammad Bidkhori

**Affiliations:** a Department of Health, Shahrekord University Of Medical Science, Shahrekord, Iran; b Department of Environmental Health Engineering, Neyshabur University of Medical Sciences, Neyshabur, Iran; c Student Research Committee, Esfarayen Faculty of Medical Sciences, Esfarayen, Iran; d Department of Public Health, Neyshabur University of Medical Sciences, Neyshabur, Iran

**Keywords:** Tobacco, Cigarette smoking, Human development index

## Abstract

This study was conducted to investigate the effect of human development index (HDI) on tobacco smoking prevalence in men and women of countries which their data about tobacco smoking were available for 2015. Pearson's correlation coefficient and linear regression were used to investigate the association between HDI and all types of smoking, particularly cigarette. Daily smoking and current smoking were used as tobacco smoking indices. The information about prevalence of tobacco smoking and HDI was obtained from the World Health Organization (WHO) website and United Nations Development Programme (UNDP), respectively. The results showed that there is no statistically significant relationship between HDI and current tobacco smoking in men (B = −0.45_CI 95%: −29.97, 29.06). However, the same association was significant for women (B = 43.87, CI 95%: 24.97–62.78). The results indicated that women in developed countries are more at risk of health effects attributed to tobacco smoking. Countries should focus on socioeconomic factors to prevent the spread of risk factors for non-communicable diseases.

**Specifications Table**

**Table:** 

Subject area	Nursing and Health profession
More specific subject area	environmental science
Type of data	Table and figure
How data was acquired	Secondary data
Data format	Raw and analyzed
Experimental factors	Linear Regression Analysis and Pearson Correlation Coefficient using STATA software were used to examine the relationship between the indicators mentioned in the abstract.
Experimental features	The relationship between the Human Development Index and the prevalence of tobacco and cigarette smoking was investigated in men and women
Data source location	Data Obtained from: World Health Organization and United Nations Development Programme
Data accessibility	Data are available from:
	http://www.who.int/tobacco/global_report/2013/full_dataset/en/
	http://hdr.undp.org/sites/default/files/2016_human_development_report.pdf

**Value of the data**

• Evaluating the effect of human development index (HDI) on tobacco smoking prevalence among men and women is required for various countries.
• In order to avoid the adverse health effect of tobacco smoking, policy-makers need to be focused on socioeconomic factors affecting smoking prevalence.
• This study indicated that in order to prevent smoking, action plans should be designed based on different age groups.
• This study showed that the lifestyle of women in developed countries is different from those in developing countries, and this increases the risk of non-communicable diseases.

## 1 Data

The data required for this study included: the prevalence of tobacco smoking among men and women, the prevalence of cigarette smoking among men and women, and human development index (HDI), (Table 1).

**Table 1:** Prevalence of tobacco smoking among men and women, the prevalence of cigarette smoking among men and women, and human development index.

Country	CTS in men	CTS in women	DTS in men	DTS in women	CCS in men	CCS in women	DCS in men	DCS in women	HDI 2015
Benin	12.5	0.7	10.4	0.5	8.8	0.2	8.2	0.1	0.485
Congo	47	1.7	33.8	1.1	28.8	0.8	24.7	0.5	0.592
Ethiopia	8.5	0.4	6.1	0.2	7.1	0.1	5.6	0.1	0.448
Kenya	20.8	1.3	16.4	0.7	17.3	0.4	14.7	0.4	0.555
Mauritius	41	3.3	31.2	1.5	36.7	2.8	29	1.1	0.781
Rwanda	21.4	4.8	17	3.7	15.2	1	11.9	1	0.498
Senegal	16.8	0.4	14.1	0.3	13.9	0.1	11.5	0.1	0.494
Canada	17.3	12.7	12.4	9	16.6	12.1	12.3	9	0.92
Costa Rica	17.7	6.5	11.1	3.9	15.6	5.8	9	3.1	0.776
Mexico	22.1	7.1	12.8	4	20.3	6.4	12.2	3.8	0.762
America	25.1	19.6	18	14	20.2	15.8	15.8	12.4	0.92
Egypt	48.9	0.3	44.2	0.2	37.6	0.1	31.9	0.1	0.691
Austria	32.2	29	24.3	22.9	30.4	27	23.8	21.9	0.893
Azerbaijan	43.5	0.3	33.2	0.2	34.2	0.3	27.3	0.2	0.759
Bulgaria	45.4	30.6	35.8	21.3	41.2	28.3	33.3	20.4	0.794
Croatia	40	33.7	35.4	28.8	36.1	30	29.7	21.9	0.827
Czech Republic	38.6	30.3	30.1	21.4	32.3	25	28.9	21.1	0.878
Denmark	19.8	19.9	15.4	15.9	17.8	18.8	12.7	11.8	0.925
Iceland	15.9	14.9	11.6	11.9	12	11.8	11.1	9.5	0.921
Ireland	26.3	23.8	20.4	18.2	22.9	20.9	17.9	16.1	0.923
Italy	28.1	19.8	23.7	16.2	27.3	19.4	22.6	16	0.887
Luxembourg	26.8	21.4	20.1	16.6	24.4	18.3	17.3	13.9	0.898
Norway	21.7	20.7	14.9	14.7	16.4	17.2	12.9	11.6	0.949
Poland	33.8	23.8	28.7	19.6	31	21.9	27.1	17.7	0.855
Sweden	19.5	19.5	10.1	11.9	12	16.3	9.3	8.9	0.913
Ukraine	48.2	13.7	43	10.2	45.2	12.8	39.8	8.9	0.743
Australia	17	13.5	14.8	12	13.4	10.9	11.9	9.3	0.939
Brunei Darussalam	30.9	2.1	24.1	1.5	25.4	1.7	19.8	1.2	0.865
China	48.7	2	42.3	1.7	43.9	1.8	38.2	1.3	0.738
Japan	34.7	11.4	29.6	9.4	33.4	10.9	28	8.8	0.903
Lao People's Democratic Republic	52.1	7.7	44.7	6.2	40	6.1	38.2	4	0.586
Malaysia	43	1	34.4	0.7	36.6	0.8	31.5	0.6	0.789
Philippines	41.5	8	31.9	5.8	37.3	6.8	29.2	5.2	0.682
Republic of Korea	42	6.2	39.3	5.3	39.6	5.9	37.6	4.9	0.901
Viet Nam	46.4	1	37.6	0.8	0.6	28.3	0.8	36.4	0.68

**Fig. 1:**
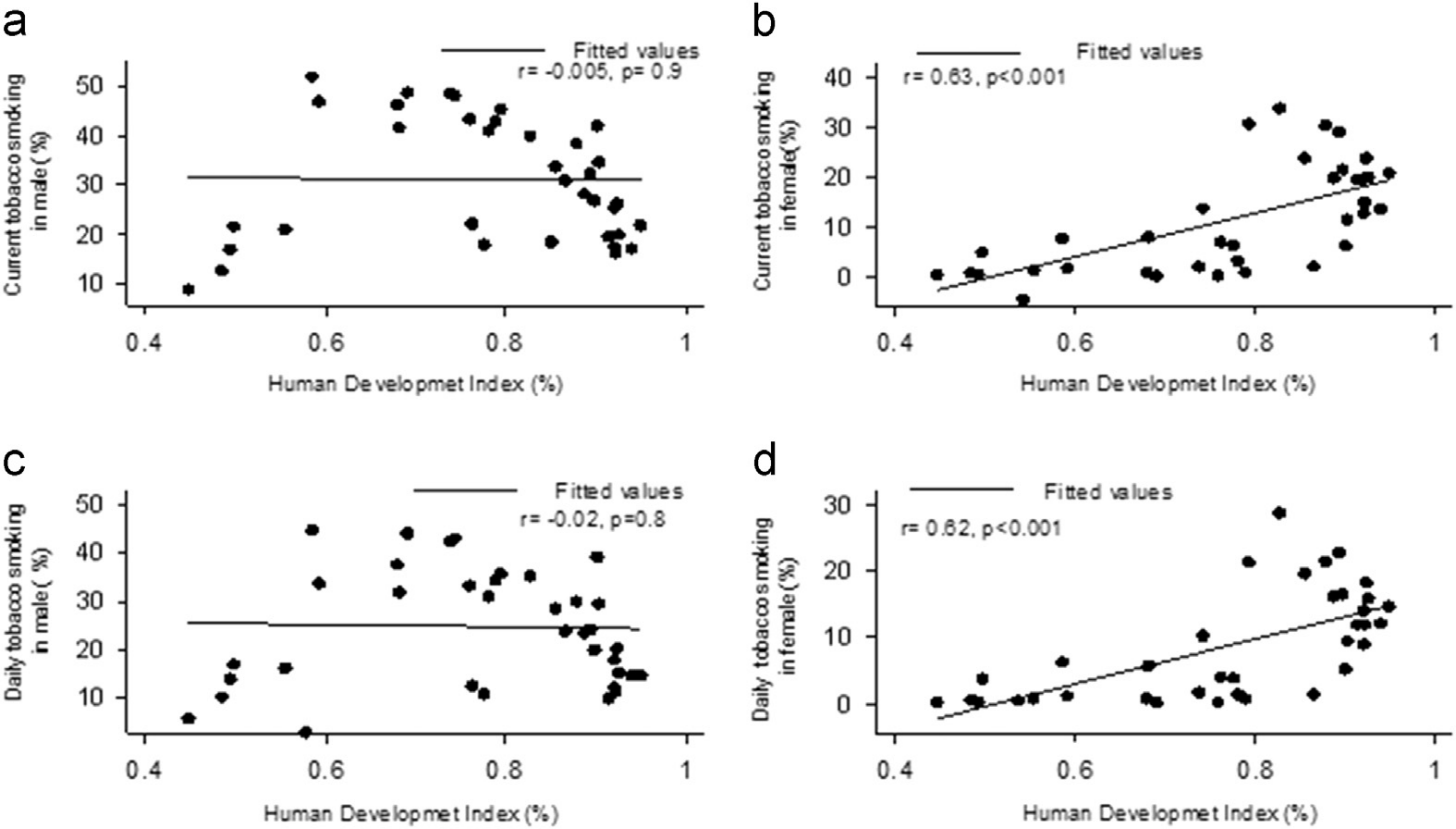
Correlation between HDI and prevalence of tobacco smoking. a: current tobacco smoking in men, b: current tobacco smoking in woman c: daily tobacco smoking in men d: daily tobacco smoking in women.

HDI showed a positive significant correlation with current tobacco smoking (CTS) (r = 0.63, *p* < 0.001) and daily tobacco smoking (DTS) (*r* = 0.62, *p* < 0.001) in women. However, the same correlations were not significant for men (*r* = -0.005, *p* = 0.9 and *r* = -0.02, *p* = 0.8, respectively), (Fig. 1).

**Fig. 2:**
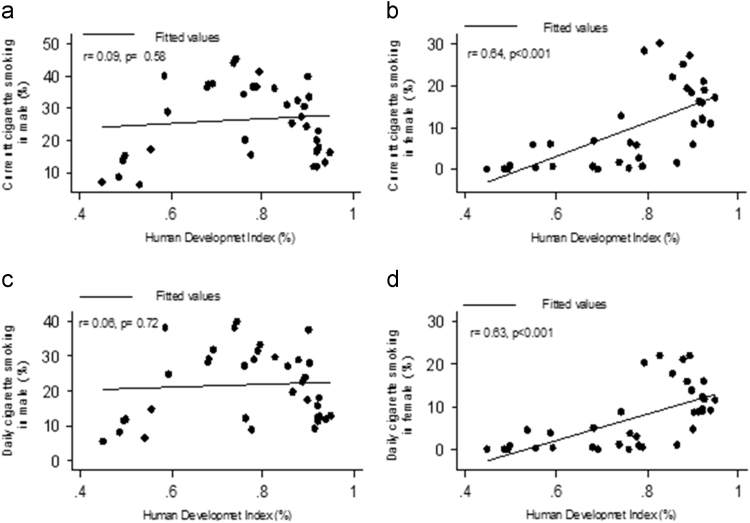
Correlation between HDI and prevalence of cigarette smoking. a: current cigarette smoking in men, b: current cigarette smoking in woman c: daily cigarette smoking in men d: daily cigarette smoking in women.

HDI was correlated positively and significantly with current cigarette smoking (CCS) (*r* = 0.64, *p* < 0.001) and daily cigarette smoking (DCS) (*r* = 063, *p* < 0.001) among women. The same correlations were not significant for men (*r* = 0.09, *p* = 0.58 and *r* = 0.06, *p* = 0.72, respectively), (Fig. 2).

Linear regression was applied to investigate the effect of HDI on the prevalence of smoking among men and women. HDI showed a significant relationship with prevalence of CTS (B = 43.87, CI 95%: 24.97, 62.78) and DTS (B = 33.74, CI 95%: 18.89, 48.59) among women, and not the prevalence of CTS (B = −0.45, CI 95%: −29.97, 29.06) and DTS (B = 3.25, CI 95%: −28.88, 24.79) for men (Table 2). Despite for men, the prevalence of CCS and DCS among women had a statistically significant relationship with HDI (Table 3).

**Table 2:** Effect of HDI on: prevalence of current tobacco smoking, prevalence of daily tobacco smoking, prevalence of current cigarette smoking and prevalence of daily cigarette smoking in men 2015.

Independent variable	Dependent variable	B	*p*-Value	95% Confidence interval
HDI				
	Current tobacco smoking	−0.45	0.97	(−29.97 to 29.06)
	Daily tobacco smoking	−3.25	0.87	(−28.88 to 24.79)
				
HDI				
	Current cigarette smoking	7.04	0.58	(−18.80 to 32.89)
	Daily cigarette smoking	4.09	0.72	(−19.49 to 27.68)

**Table 3:** Effect of HDI on: prevalence of current tobacco smoking, prevalence of daily tobacco smoking, prevalence of current cigarette smoking and prevalence of daily cigarette smoking in women 2015.

Independent variable	Dependent variable	B	*p*-Value	95% Confidence Interval
HDI				
	Current tobacco smoking	43.87	< 0.001	(24.97– 62.78)
	Daily tobacco smoking	33.74	< 0.001	(18.89–48.59)
				
HDI				
	Current cigarette smoking	40.54	> 0.001	(23.61–57.46)
	Daily cigarette smoking	30.72	< 0.001	(17.51–43.93)

## 2 Experimental design, materials and methods

### 2.1 Study countries description

Tobacco smoking is introduced as a major preventable cause of death and risk factor for cardiovascular diseases [1], [2], [3], [4], [5]. Human development index is combined of three parts, including life expectancy at birth, mean years of schooling, and gross national income per capita [6], [7], and its value is between 0 and 1 [8]. The information about the prevalence of tobacco smoking and HDI was acquired from the World Health Organization (WHO) and United Nations Development Programme (UNDP) websites, respectively [8], [9]. Due to lack of information for a constant baseline year, only countries were included in this study that their prevalence of tobacco smoking was reported for 2015.

### 2.2 Analytical procedures

In this study, Pearson's correlation and linear regression were used to analyze the possible correlation between indices and the relationship between variables, respectively. All the statistical analyses were performed using STATA 14.
